# Effect of Target Composition and Sputtering Deposition Parameters on the Functional Properties of Nitrogenized Ag-Permalloy Flexible Thin Films Deposited on Polymer Substrates

**DOI:** 10.3390/ma11030439

**Published:** 2018-03-17

**Authors:** Waheed Khan, Qun Wang, Xin Jin

**Affiliations:** College of Materials Science and Engineering, Beijing University of Technology, Beijing 100124, China; jinxin1118@emails.bjut.edu.cn

**Keywords:** magnetron sputtering, transmission electron microscopy (TEM), X-ray photoelectron spectroscopy (XPS), permalloy, surface free energy

## Abstract

We report the first results of functional properties of nitrogenized silver-permalloy thin films deposited on polyethylene terephthalic ester {PETE (C_10_H_8_O_4_)_n_} flexible substrates by magnetron sputtering. These new soft magnetic thin films have magnetization that is comparable to pure Ni_81_Fe_19_ permalloy films. Two target compositions (Ni_76_Fe_19_Ag_5_ and Ni_72_Fe_18_Ag_10_) were used to study the effect of compositional variation and sputtering parameters, including nitrogen flow rate on the phase evolution and surface properties. Aggregate flow rate and total pressure of Ar+N_2_ mixture was 60 sccm and 0.55 Pa, respectively. The distance between target and the substrate was kept at 100 mm, while using sputtering power from 100–130 W. Average film deposition rate was confirmed at around 2.05 nm/min for argon atmosphere and was reduced to 1.8 nm/min in reactive nitrogen atmosphere. X-ray diffraction, X-ray photoelectron spectroscopy, scanning electron microscopy, vibrating sample magnetometer, and contact angle measurements were used to characterize the functional properties. Nano sized character of films was confirmed by XRD and SEM. It is found that the grain size was reduced by the formation of nitride phase, which in turns enhanced the magnetization and lowers the coercivity. Magnetic field coupling efficiency limit was determined from 1.6–2 GHz frequency limit. The results of comparable magnetic performance, lowest magnetic loss, and highest surface free energy, confirming that 15 sccm nitrogen flow rate at 115 W is optimal for producing Ag-doped permalloy flexible thin films having excellent magnetic field coupling efficiency.

## 1. Introduction

Recent trend shift from microelectronics to nano-electronics is demanding the development of flexible nano-thin films suitable for high-tech flexible technologies. The evolution from traditional coatings to flexible thin films are making substantial scientific and viable impact by aiding the emergence of, flexible displays [1], flexible thin film transistors [2], flexible and thin film solar cells [3,4], flexible smart textiles [5], flexile ferroelectric random access memories [6], magneto impedance sensors [7], and flexible photovoltaics [8]. High quality flexible thin films are typically achieved by depositing Inorganic materials, such as metals, functional metals, and nitrides, deposited onto polymer substrates via direct current (DC)/radio frequency (RF) plasma sputtering [9,10], co-sputtering [11], and spin coating [12].

Unique structures with a high density of interfaces, granular magnetic materials are comprised of nano sized ferromagnetic particles that are distributed in an immiscible medium [13]. Cenospheres with thin coats of Cu are produced by vibration-assisted magnetron sputtering with significant prospective for the production of novel varieties of metal matrix syntactic foams, along with optimized alternatives of conventional materials of the same type [14]. Thus, gives rise to a variety of enhanced properties that are of fundamental interest and technological importance [15]. The research that was conducted on granulated materials was mainly dedicated on systems in which the granules are elemental metals [15]. Since alloys shows remarkable functional properties, it is of interest to explore granular alloy thin film systems [16]. Magnetic anisotropy in such systems allow for regulating electronic spin currents through magnetically doped nanometer structures by an external magnetic field is of great technological interest [17].

Amongst the materials playing a key role in these nanostructures are noble-metal-free alloy system of Ni and Fe. Permalloy alloy with composition of Ni_80_Fe_20_ is a well-known soft magnetic alloy with limited-dimensional structures and forms a FCC structure of the type Ni_3_Fe. Owing to their outstanding soft magnetic properties, including low coercivity (Hc) and energy loss, high saturation magnetization (Ms), and high permeability (μ), they are widely used in electromagnetic microwave absorption, magnetic recording devices, magnetic resonance imaging, and sensors [18,19,20,21,22]. Obviously, the above-mentioned magnetic properties of NiFe alloys are closely related to the processing parameters, composition, and phase configuration.

Reactive magnetron sputtering is convenient and accurate technique used to introduce the nitrogen into depositing thin film in order to adjust its structural, magnetic, and electric properties [23]. NiFe nitrides can be deposited using an RF sputtering technique for varying nitrogen flow in the range of 5–30% and to tune the various physical and magnetic properties of NiFeN thin films. Different phases of FeN films can be obtained through changing N_2_ fraction in a mixture of N_2_/Ar gas flow [24]. A reduction in saturation magnetization was established with the increase of nitrogen flow during sputtering [25,26,27]. It is also well known that the incorporation of nitrogen by sputtering processes on metals induces the formation of amorphous or fine-grained structures [28]. However, a detailed investigation of magnetic properties with an increase in nitrogen content has not been performed yet on flexible thin films, as reactive nitrogen sputtering may induce structural and magnetic changes. It is clear that processes, which occur at the surface during sputtering, critically affect the functional properties of as-deposited films [29]. Therefore, parameters such as grain size, the binding energies of surface atoms, and sputtering power will control the stoichiometry [30,31]. Permalloy thin films grown by electrodeposition display vortex magnetization with arbitrary chirality and polarity, which is described in terms of dipolar interface minimization [32].

The use of alloying and doping of magnetic thin films are used to fine-tune the properties for practical applications. These heterogeneous alloys can be designed by co-depositing [33] the two immiscible metallic components, one magnetic, the other nonmagnetic (e.g., Co. and Ag) on a substrate. Owing to their immiscibility, the components are likely to segregate ensuing the formation of nonmagnetic matrix with small magnetic precipitates embedded in it [34,35,36,37]. After replacing a minor amount of Ti ion with Fe, extremely enhanced ferroelectric characteristics were obtained successfully, while the thin films were annealed in nitrogen, air, and oxygen atmospheres [38]. Thickness dependence, which is mainly due to deposition time or power variations of the magnetic hysteresis observed in NiFeAg heterogeneous alloy films at ambient temperature onto glass substrates [39]. Higher charge voltage could expedite AlCrN coatings having a compact columnar microstructure by means of modulated pulsed power magnetron sputtering (MPPMS), with altered power pulse parameters [40]. In principle, if the particle size dispersal rests constant with thickness, the GMR in heterogeneous alloys would not be affected. GMR effect is a very important phenomenon that happens in magnetic materials going from thin films to nanoparticles over multilayered permanent magnets [41]. Copper-permalloy films were deposited by co-sputtering, inducing Cu into the permalloy lattice results in very resilient spin scattering and alters their chemical, structural, magnetic, and electrical properties [42]. Interfacial scattering affects the magnetoresistance of heterogeneous FeCoAg films and the magnetic couplings between the particles are considerably decreased because of the coalescence of the magnetic species [43,44]. Ag concentration in the as-deposited NbCN-Ag thin films were accomplished by adjusting Ag target power to tune the functional properties [45].

In this paper, we report the first results on an experimental study that was carried out on the as-deposited Ag-Permalloy thin films deposited on polymer substrates using mosaic alloy target at room temperature. The effect of silver content, sputtering power, and nitrogen flow rate on functional properties is investigated systematically. The aim of this study is to establish a scientific correlation between structural, magnetic, optical, and surface properties evolved. We have deposited Ni-Fe-Ag films, having a fixed Fe concentration and varying Ni and Ag concentrations applying increasing sputtering power.

## 2. Experimental Method

Three distinct types of NiFeAg granular thin films were prepared by DC reactive magnetron sputtering on flexible polyethylene terephthalic ester (PETE) substrates. Conventional glass substrates are brittle and non-deformable. In order to develop flexible components from soft magnetic thin films, polymer substrate is most suitable. Further work of mechanical testing is in progress to check the flexibility limits of thin films. Two specially prepared mosaic targets of silver-doped Permalloy (Ni_76_Fe_19_Ag_5_ and Ni_72_Fe_18_Ag_10_) were used in the 5n (99.999%) pure Ar gas atmosphere. The target was composed of Fe, Ni, and Ag. As Ag forms no compounds with Fe, hence the target is believed to be composed of elemental sectors, making it a mosaic formation. Samples 1–3 were deposited by the sputtering of Ni_76_Fe_19_Ag_5_ target, and samples 4–6 by using Ni_72_Fe_18_Ag_10_ target under argon plasma atmosphere. While the samples 7–9 were deposited using reactive sputtering of nitrogenized Ni_72_Fe_18_Ag_10_ using a mixture of N_2_+Ar. As for the lower magnetization moments and not appreciable P-loss properties, it was decided not to deposit thin films of Ni_76_Fe_19_Ag_5_ under a reactive atmosphere. PG-32B, polymer substrates were provided by lucky corporation China (Beijing, China) having uniform thickness equals to 188 μm thick. Firstly, sputtering power was increased from 100–130 W for different film structure and thicknesses, while keeping the substrate at the system ambient temperature. Secondly, the nitrogenized thin films were grown in the gas mixture of N_2_+Ar, using varied gas nitrogen flow rates, while keeping the sputtering power constant at 115 W. Two independent mass flow controllers precisely controlled the Ar and N_2_ flow rates. Aggregate flow rate and the total pressure of Ar+N_2_ mixture was 60 sccm and 0.55 Pa, respectively. A base pressure of about 4.47 × 10^−5^ Pa was achieved in the sputtering chamber and the target was sputtered for few minutes at 100 W to eliminate surface contaminations prior to the deposition. All of the thin films in argon atmosphere were sputtered for 100 min, while the sputtering time in reactive nitrogen atmosphere was 110 min. Distance between target and the substrate was kept at 100 mm. The substrate holder was rotated clockwise at 10 rpm to ensure the homogeneity of deposited samples. Deposition processing parameters and sample ID’s are given in Table 1. Thickness of as-deposited thin films was measured by a surface profilometer (VEECO Dektak ADP-8, BJUT, Beijing, China).

The structures of the films were examined using X-ray diffraction (XRD) with Cu Kα radiation (λ = 0.15406 nm) using a current of 30 mA and voltage of 40 kV (Shimadzu, Kyoto, Japan, XRD 7000). Continuous scan was acquired with drive axis = θ–2θ in the range of 20° to 80°. The in-plane static magnetic properties of thin films were measured using a vibrating sample magnetometer equipped with MicroSense easy VSM software 9.13wa in magnetic fields up to 2.1 T. ThermoScientific ESCALAB 250x (Thermofisher, Stockholm, Sweden), X-ray photoelectron spectroscopy (XPS) was used to determine the chemical binding of atoms and composition. Monochromatic source of Al Kα radiation with energy resolution of 0.5 eV and voltage approaching to 1500 eV was applied [9,10]. Argon ion (Ar^+^) sputtering was used to clean the thin films surface from contamination and oxides. For power loss (PL) evaluation, thin film samples (20 × 40 mm^2^) were accurately positioned in the mid of microstrip line (MSL), having a characteristic impedance of 50 Ω. Two ends of the MSL are tightly coupled to a vector network analyzer (CETC, AV36850A, Agilent, Santa Clara, CA, USA), which can measure scatter parameter in the blue tooth frequency range of 1 MHz to 3 GHz [46,47].

Two calibrated near filed probes (RF 400-1/2, Agilent, Santa Clara, CA, USA) were used to investigate magnetic field coupling efficiency, first as a transmission coil linking to signal generator (Agilent, Santa Clara, CA, USA, E8257D) and the second as a receiver coil joining with EMC analyzer (Agilent, E7405A). After an alternating current (AC) was allowed to pass from the transmission coil, magnetic lines of field were produced and were passed through the receiving coil. Thus, creating an induced electromotive force (EMF) around it, which in turn, formed a magnetic field couple. Resultant values were measured from EMC receiver by attaching the thin film samples to the transmission coil as a backplane and efficiency of magnetic field couple was measured. Complex permeability of the samples was measured by Agilent-4396B network spectrum impedance analyzer equipped with a dielectric material test fixture (Agilent, 16453A). Toroidal shaped samples with outer and inner diameter of 8 mm and 3 mm, respectively, were cut from the thin films with an especially designed alloy-steel cutter.

Surface free energy at ambient temperature of thin films was measured by contact angle measurements; details are given elsewhere [9]. Scanning electron microscope (JSM-7610F, JEOL, Beijing, China) was used to examine the surface morphology and elemental analysis by using aperture angle control lens, which automatically optimizes the spot size at both high and low currents.

## 3. Results and Discussion

### 3.1. X-ray Diffraction Phase Structure (XRD)

**Figure 1:**
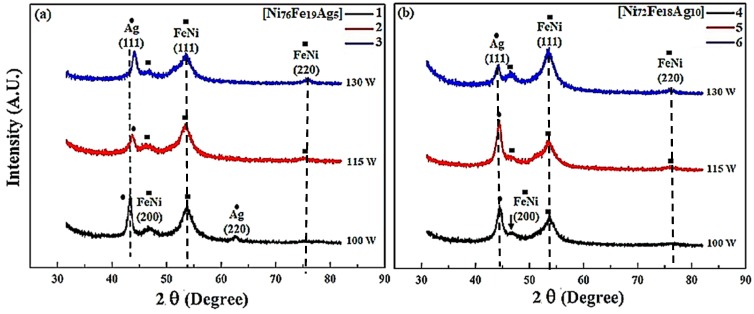
X-ray diffraction (XRD) pattern of thin films using different target compositions (**a**) Ni_76_Fe_19_Ag_5_; (**b**) Ni_72_Fe_18_Ag_10_, and sputtering power (samples 1 and 4 = 100 W: 2 and 5 = 115 W: 3 and 6 = 130 W).

Figure 1 is the XRD pattern of flexible thin films illustrating the effects of composition and sputtering power on the structure of the as-deposited samples. Figure 1a shows the pattern of samples 1–3 deposited using Ni_76_Fe_19_Ag_5_ target composition. As the sputtering power is increased from 100–130 W for sample 1–3, respectively, the broadening of reflection and shift toward a higher angle is observed and indicated as a vertical line. Evident diffraction peaks of pure silver crystal faces are at ±42° (111) in all of the samples and 62° (220) in sample 1. The Ag (111) peak depicts a rocking curve breadth of 4.5° in the as-deposited films. The intensity of the Ag (111) peak decreases at 115 w, and then increases at 130 w sputtering power, depicting that line breadth decreases as the sputtering power increases, showing that smaller crystallites of Ag are formed and the NiFe lattice is further strained due to coherent precipitation of Ag particles. As the grain size decreases, there is a substantial rise in the volume fraction of grain boundaries or interfaces. This feature strongly effects the chemical and physical properties of the thin films. Specifically, a decrease in the grain size results in better soft-magnetic properties. Results indicate the presence of two FeNi peaks at 52° and 46°, corresponding to (111) and (220) planes, respectively, in sample 1, while there is a weak peak at 75° (220) in samples 2 and 3.

Strong (111) reflections were perceived demonstrating highly (111) textured thin films with FCC structures of both NiFe and Ag. Secondly, the NiFe (200) and (220) line intensity increases with the increasing deposition power, indicating the breakdown of the (111) texture of the film at around 130 W. The positive role of the increase of sputtering power is taking place in certain ranges, a too high sputtering power could lead to too energetic ad-atoms, which could induce morphological and/or structural defects. Figure 1b shows the XRD patterns of samples 4–6 that were prepared by using target composition Ni_72_Fe_18_Ag_10_ at an increased sputtering power from 100–130 W, respectively. With the decrease of Ni content and the increase of Ag, reflections shifted slightly towards each other for all of the samples, indicating either NiFeAg alloying or strain from coherent NiFe/Ag interfaces [13,48].

Evident diffraction peaks of Ag (111) in samples 4–6 are at ±43.5° with slightly higher intensities. Assuming alloying between NiFe and Ag, the peak shift corresponds to Ag diluted in NiFe. Results indicate the presence of two FeNi peaks at 54° and 47° corresponding to (111) and (220) planes, respectively, in sample 4, while there is a weak peak at 76° (220) in samples 2 and 3. The NiFe (111) peak of sample 6 is strongest at high processing power (130 W), but appears only as a slight shoulder at lower angle and power (100 W), representing a very uniform dispersion of NiFe within the Ag matrix. The peak breadth of both, NiFe and Ag reflections are broad, indicating either non-uniform strains, nano particle sizes, or well-distributed concentrations of NiFeAg alloy. During sputtering the ad-atoms possess energy nearly few tens of eV, and in the course of condensation onto the substrate, ad-atoms are quenched and may form an amorphous or fine-grain structure. Ag peaks were compared with PDF# 04-0783, 01-0870719, 65-2871, and 87-0597, while NiFe reflections were studied in comparison with PDF #47-1405, 37-0474, and 03-1109.

**Figure 2:**
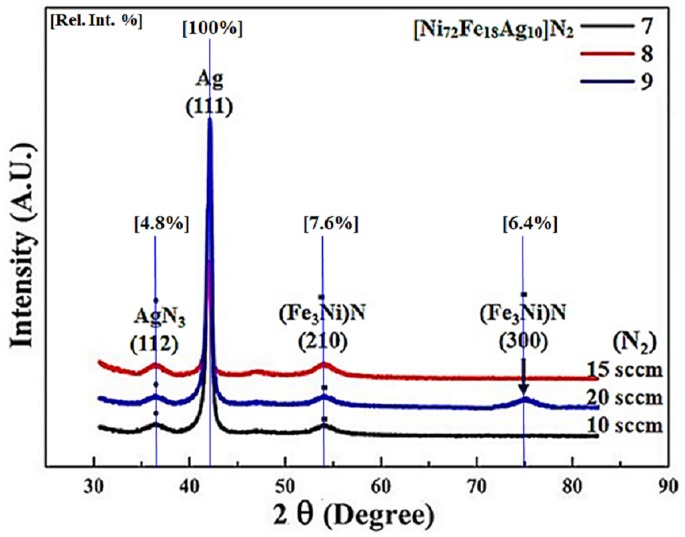
XRD pattern of nitrogenized thin films using target composition Ni_72_Fe_18_Ag_10_, under Ar+N_2_ gas mixture with different amounts of reactive nitrogen during sputtering.

Figure 2 shows the XRD patterns of samples 7–9 prepared by using target composition Ni_72_Fe_18_Ag_10_ under Ar+N_2_ gas mixture with varying nitrogen flow rates at a constant sputtering power of 100 W. Relative intensity (%) is shown as the vertical lines in the XRD pattern. Gas mixture in the sputtering process is the most important processing parameter, and it has a substantial impact on the composition and the phase structure of the films [49]. Characteristic sharp peak of cubic structure of Ag (111) as a result of larger grains is depicted at 41°, but there are no additional peaks that are associated to that specific structure. When considering the breadth of the peak, it is a convincing inference that this peak specifies crystalline behavior. The peak of orthorhombic AgN_3_ (112) corresponds to the value of 2θ = 36.5° [50]. The higher intensity of an Ag phase peak is due to the (111) preferred orientation. Also, the FeNi and (Fe_3_Ni) N phases are not fluorescent and are not easy to detect by XRD. Hence the signal of FeNi and (Fe_3_Ni) N phases are lower than the more fluorescent Ag phase.

Nitrogenized Ag-Permalloy thin film reveals cubic nitride of NiFe belonging to space group 221 having calculated density of 7.33 g/cm^3^. Sample-7 at 10 sccm flow rate of nitrogen shows the major peak of cubic (Fe_3_Ni) N (210) at 54°.

At an increased nitrogen flow rate of 15 sccm, sample 8 shows the peaks with higher intensity values exactly at the same position without any shift, suggesting that the increase of nitrification does not affect the lattice parameters. Also, the line breadth of the Ag (111) peak is observed not to increase as smaller grains of Ag are formed and lattice is not strained due to coherent precipitation of NiFe particles. Together, when sputtered by means of low-Z reactive nitrogen ions, they may occupy interstitial sites in the unit cell of sputtered species, instigating an alteration of the unit cell. A combined outcome of these conditions may lead to a nanocrystalline structure of the as-deposited film besides constraining the long-range ordering. As for Fe the structure is BCC and for NiFe it is FCC. The possibility of occupying the interstitial sites in the BCC and FCC crystal system is dissimilar because of different close packing. Figure 2 shows a cross when nitrogen flow increased to 20 sccm, another (Fe_3_Ni) N peak appears at 75° along with all the other peaks depicted at lower nitrogen flow rates. Peaks height (intensity) is lower than the intensities of sample-8 due to over-nitrification of the thin film, PDF#03-065-7529. This suggests that increase of nitrogen above a certain limit during sputtering affects the long-range order of the thin films. The interstitial site in the FCC NiFe alloy is bigger than that in the BCC Fe. While both are smaller as compared to the atomic radius of a nitrogen atom (0.075 nm) and since existing space in a FCC structure is larger, it accepted more nitrogen atoms to be incorporated. As the nitrogen partial pressure is increased, the broadening of the X-ray diffraction is a clear indication of the nanocrystalline nature of the films because the incorporation of nitrogen favors the growth of smaller crystallites [28]. At lower nitrogen partial pressures, nitrogen ions do not react with Ag, Fe, Ni, or NiFe, and nitrogen is integrated in the interstitial sites, causing an expansion of the unit cell. At intermediary nitrogen pressure, a chemical reaction amongst nitrogen, and Fe, Ni, or NiFe is possible, causing the disposition of nitride phases. At higher nitrogen pressures, deformation of the formed nitride phase begins and the resultant structure is again nanocrystalline.

### 3.2. (XPS) Binding Energy and Surface Composition

Ernest Rutherford (1914) equation is widely used to estimate the electron binding energy of discretely emitted electrons, as the energy of an X-ray with particular wavelength is known (Al Kα X-rays, Ep = 1486.7 eV). Calculated kinetic energies is according to Equation (1):

(1) KE=hv−BE−ϕs

where *hν* is the energy of the photon, *BE* is the binding energy of the atomic orbital from which the electron originates, and *φs*, is the work function dependent on material and the spectrometer. The wide scan of thin films depicts very small O1s and C content, which are due to adsorbed CO and/or CO_2_, formed on the surface. Argon ion (Ar^+^) sputtering was used to clean the thin films surface.

**Figure 3:**
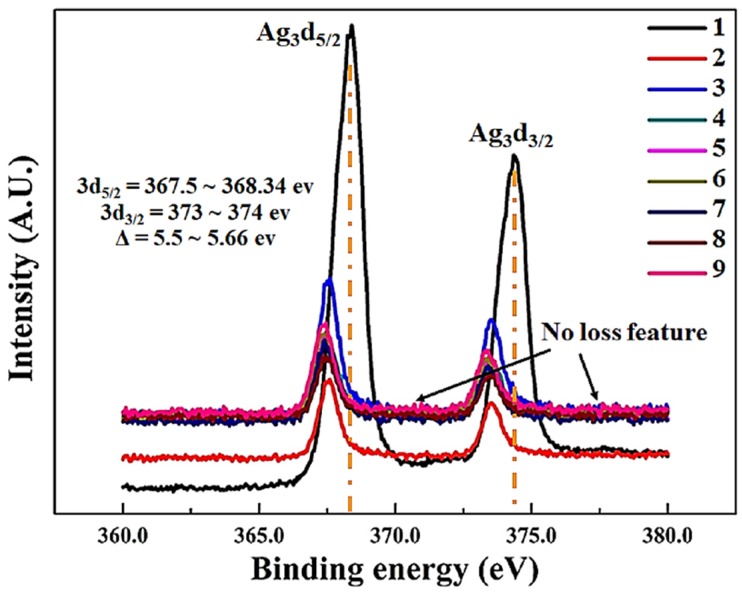
High-resolution X-ray photoelectron spectroscopy (XPS) Ag3d spectrum of thin films 1–9.

Figure 3 shows the XPS spectra of Ag_3_d for thin film samples (1–9) that are deposited on PETE substrate. The binding energy of silver in sample-1 increases consistently with the increase of intensity and the binding energy of 374 eV is related to the stable Ag^+^. Stability of silver is mainly due to the presence of Ni and Fe atoms in nearby locations. The large difference between electro negativities of Ag with other elements (Ni and Fe) generates the change of electron distribution at the interface is due to NiFe deposition.

Samples 2–9 have almost same range of binding energies, while the intensities of samples 3, 6, and 9 are higher due to increased sputtering power, more Fe and Ni atoms tends to appear at the surface, while keeping the overall stoichiometry same. In addition silver compounds are typically X-ray sensitive. Ag3d region has well split spin-orbit components (Δ = 5.5~5.66 eV). Peaks have asymmetric “peak shape” for thin films 1–3 and small binding energy shifts towards lower values for samples 4–9 Ag_3_d peaks broaden with respect to increase of Ag percentage. Loss features were not observed on both sides of binding energy of each spin-orbit component [51,52].

**Figure 4:**
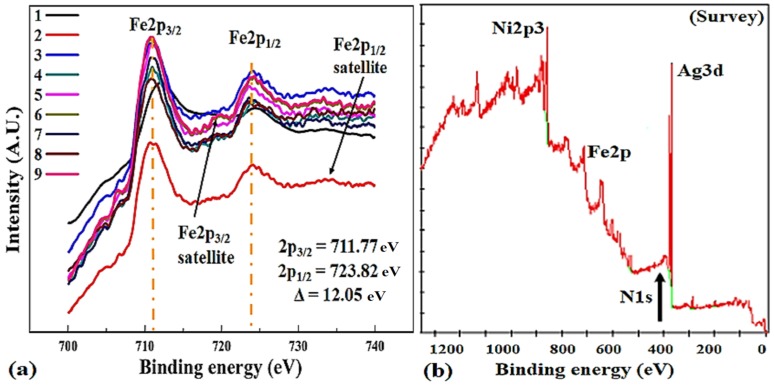
(**a**) High-resolution XPS Fe2p spectrum of thin films 1–9; (**b**) shows the survey and small peak of N1s.

Figure 4a shows the Fe2p spectra of samples 1–9 and a close view of the Fe2p_2/3_ region, indicating the possible states of Fe. The binding energies of 711.77 and 723.82 eV are related to Fe2p_3/2_ and Fe2p_1/2_ regions, respectively. The binding energy distinguishes the chemical environment that the atom is be subjected to.

Broad satellite peaks depicts the existence of oxidized Fe^2+^ and Fe^3+^ states in samples 2, 5, and 8 that are deposited at an optimal power of 115 W [53]. Atomic sensitivity factor (ASF) at 90° of x-rays for Fe2p is 2.686, much higher than 0.477 for N1s, this can be used to compute the atomic percentages in the thin films. Slightly higher binding energy of sample 1 (712.2 eV) is possibly due to electron-deficient Fe^2+^ sites, which are created by the breaking of Ni-Fe bonds. Fe2p region shows significantly separated spin-orbit components (Δ = 12.05 eV) and peaks have asymmetric shape, while Fe2p_1/2_ spectrum shows multiplet splitting [54]. N1s spectrum and survey is displayed in Figure 4b for samples 7–9.

**Figure 5:**
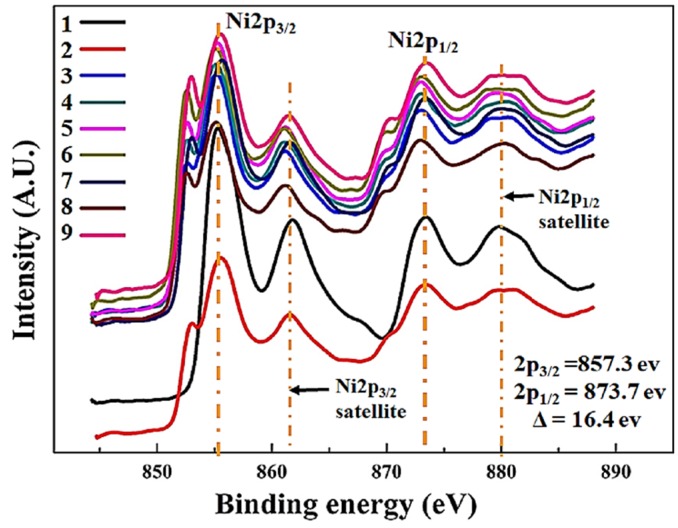
High-resolution XPS Ni2p spectrum of thin films 1–9.

Figure 5 shows the Ni2p region spectrum of samples 1–9 and the presence of Ni species in different states. The peaks of Ni2p_3/2_ region located around 857.3 and 861.4 eV can be assigned to metallic Ni^2+^ and Ni satellite, respectively [55]. Two major Ni2p peaks has significantly split spin-orbit components (Δ = 16.4 eV). Ni XPS spectrum has complex shape showing a mixture of core level and satellite features. Satellite features not to be confused with oxidized nickel peaks. NiFe compounds can also have complex, multiplet-split peaks [53].

The intensities varies with sputtering power and samples 3, 6, and 9 have the highest in the respective set of experiments, while sample 8 attained maximum intensity, which was deposited at 15 sccm nitrogen flow rate. ASF for nickel, 3.653, is higher than Fe making it easier and more accurate for determining the atomic percentile in the thin films. Table 2 shows the composition (at %), binding energies, ASF, oxidation state and separation values between peaks during XPS at high vacuum.

The sample compositions were measured during XPS using pass energy of 100 eV and spot size of 500 μm, and were confirmed by EDS analysis in scanning electron microscope. The composition of thin films was found to be very close to the target compositions as mentioned in Table 1 and Table 2. Although we have tried to maintain the ternary concentration Ni_76_Fe_19_Ag_5_ (samples 1–3) and Ni_72_Fe_18_Ag_10_ (samples 4–9) stoichiometry, we have found that this stoichiometry is slightly broken during sputtering under N_2_+Ar atmosphere. N1s peaks correspond to nitride formation in samples 7–9 and endorses the XRD results in principal. Higher binding energy states of nitrogen are thermodynamically unstable and decay rapidly with Ar+ sputtering, even at low beam energies [57].

No traces of individual Ni or Fe regions were found, indicating that the flexible thin film samples remains as silver-doped Permalloy. The inaccuracy in all of the peak positions is estimated to be 0.05 eV.

### 3.3. (VSM) Static Magnetic Properties

**Figure 6:**
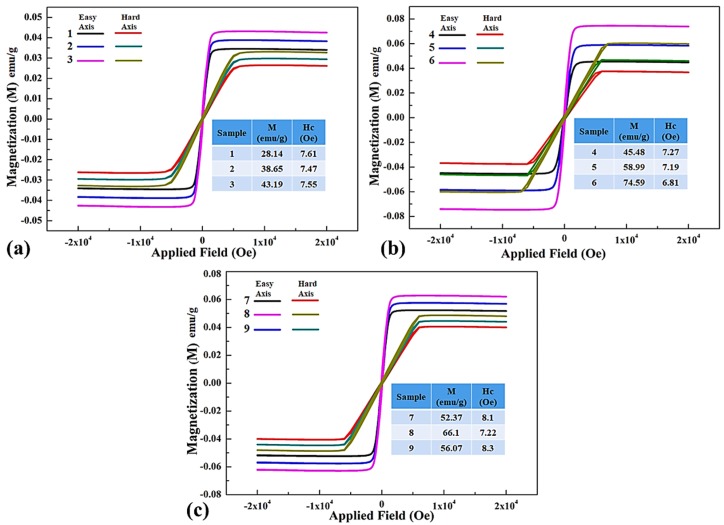
Room temperature magnetization measurements at 2.21 T (**a**) Ni_76_Fe_19_Ag_5_ thin films prepared with Ar gas using different sputtering power; (**b**) Ni_72_Fe_18_Ag_10_ thin films prepared with Ar gas using different sputtering power; and (**c**) Ni_72_Fe_18_Ag_10_ thin films prepared with N_2_+Ar gas mixture using different nitrogen flow rate.

Static magnetic properties of as-deposited flexible thin films were measured by using vibrating sample magnetometer at room temperature in magnetic fields up to 2 × 10^4^ oersteds (Oe), as shown in Figure 6.

The hysteresis loops were measured along easy and hard axis magnetization of thin films with respect to the applied magnetic field. All of the samples depict representative soft magnetic characteristics with reasonably small values of coercivity.

Figure 6a shows the hysteresis loops of samples 1–3 that are deposited at varying power during sputtering with Ag equals to 5% in permalloy. The resultant easy axis magnetizations (M) are 28.14 emu/g, 38.65 emu/g, and 43.19 emu/g for the composites with the corresponding power of 100, 115, and 130 W, respectively. These values are comparable to the 25–45 emu/g for permalloy epitaxial thin films grown on (001) MgO substrates [58]. The range of M increased with increasing the deposition power because of a steady increase in film thickness from 180 nm to 236 nm. Coercivity of samples is low from 7.61–7.55 Oe of nano structured thin films,, as values are also in agreement with literature [59]. While the thickness increases and the average size D of magnetic particles is reduced to nano level, the interchange coupling between magnetic particles will occur and forces magnetizations of particles in a parallel line. This results in the reduction of magnetic anisotropy and vanishing the demagnetization effect. Hence, the average coercivity H_c_ of the film decreased due to that energy loss that is associated with hysteresis. The coercive force varies weakly and shows slight reliance on the deposition rate rather than of thin film deposition parameters. Relationship of coercivity and saturation magnetization was studied in magnetization model [60] for a soft materials, and also was proposed by Alben et al., as follows in Equation (2) [61].

(2) $$M_{s}A^{3}\;=\;0.64\left(\frac{{K_{1}}^{4}D^{6}}{H_{c}}\right)$$

It is confirmed in XPS results that there is no loss component in thin film samples 1–3, suggesting that silver is present as a simple dilutent only [59]. Figure 6b shows the hysteresis loops of samples 4–6 deposited with varying power during sputtering having an increased Ag content of 10%. The resultant easy axis magnetizations (M) are 45.48 emu/g, 58.99 emu/g and 74.59 emu/g for the samples with the corresponding film thicknesses of 185, 196 and 242 nm respectively. Sample-6 have the highest magnetization value of 74.59 emu/g and is pity much close to the magnetization, 82 emu/g of pure Ni_81_Fe_19_ permalloy [62], along with lowest coercivity of 6.81 Oe. It seem that the magnitude of the dispersion of local magnetic isotropy is contributing to magnetization [63] of the samples as a relative portion of Fe is increased with the decrease of Ni content in the thin films due to formation of ferromagnetic particles in the heterogeneous film. Figure 6c shows the static magnetic properties of samples 7–9 that were deposited using target composition Ni_72_Fe_18_Ag_10_, keeping the sputtering power constant at 115 W. The thin films were produced under N_2_+Ar atmosphere with an increased nitrogen flow rate from 10–20 sccm. The resultant easy axis magnetizations (M) are 52.37 emu/g, 66.1 emu/g, and 56.07 emu/g, which are lower than the samples that are deposited under argon gas alone.

Sample-8 has highest magnetization under N_2_+Ar gas mixture depicting N_2_ = 15 sccm as optimal flow rate for depositing silver doped permalloy flexible thin films. At an increased flow rate of 20 sccm, the magnetization decreased to 56.07 emu/g with the increase of coercivity because of following reasons. Firstly, the presence of more Ag produced the thin films with a coercive field higher than that for the samples with lower Ag, due to the non-magnetic nature of Ag. Secondly, it is probably due to the formation of AgN_3_ phase, as evident in Figure 2 also and leads to magnetic losses [64]. Sample-9 is sputtered with more nitrogen, it adds in the interstitial positions in FCC NiFe and constrains the magnetic ordering of the thin film, and this causes a reduction in the magnetic moment. Moreover, the occurrence of two peaks of paramagnetic (Fe_3_Ni) N, as determined by XRD, leads to the increase of coercivity. It is also observed that the reduction of the average crystallite size as well as the presence of small amount of paramagnetic phase causes an important change in the magnetization reversal mechanism. Thin film of sample-9 deposited at 20 sccm of nitrogen flow, bears the highest coercivity of 8.3 Oe. It cannot be explained with only the incorporation of nitrogen in the film favors the growth of larger crystallites, and is probably related to the small amount of paramagnetic nitride formation [28].

### 3.4. (Db Loss Factor) Power Loss of Thin Films

**Figure 7:**
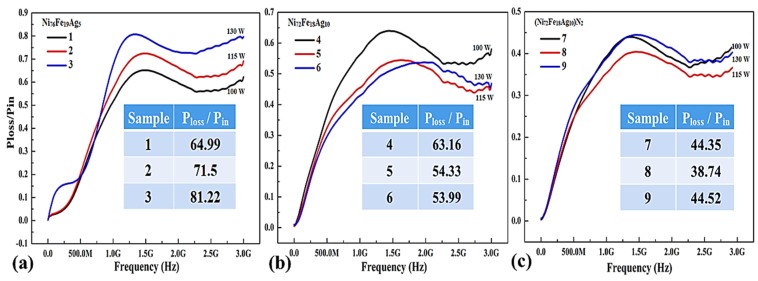
Power loss for flexible thin films (**a**) Ni_76_Fe_19_Ag_5_ thin films deposited using Ar and varying sputtering power; (**b**) Ni_72_Fe_18_Ag_10_ thin films deposited using Ar and different sputtering power; and (**c**) Ni_72_Fe_18_Ag_10_ thin films prepared with N_2_+Ar gas mixture using different nitrogen flow rate.

Figure 7 shows Power absorption characteristics as a function of frequency for silver-doped permalloy flexible thin films. Power loss functionality is crucial to subdue detrimental high-frequency electromagnetic interference in highly integrated electronic components functioning in the 1–3 GHz blue tooth range. The value of PL step up with frequency and deposition power, as estimated from their Scattering-parameters (*S*_11_ and *S*_21_), as given by Equation (3).

(3) $$\frac{P_{loss}}{P_{in}}=1-10^{\frac{S_{11}}{10}}-10^{\frac{S_{21}}{10}}$$

Figure 7a shows the effect of sputtering power on PL values for the Ni_76_Fe_19_Ag_5_ composition films. Power loss increases with an increasing thickness due to sputtering power during deposition. The increase of the sputtering power results in an increased kinetic energy of the sputtered atoms, which makes it easier for more atoms to reach the substrate. Consequently a higher deposition rate and film thickness. Sample-3 deposited at 130 W have maximum transmission loss value of 0.81, which means that 81% of input power of noise current is absorbed by the sample [65]. Figure 7b shows the effect of sputtering power on PL values for the Ni_72_Fe_18_Ag_10_ composition films. Sample-4 deposited at 100 W have similar thickness as compared to sample-1, so as the similar power loss values. Samples-5 & 6 have lower values of around 54% transmission loss. Increased magnetization, improved magnetic permeability, and magnetic loss can be achieved in gigahertz frequencies above Snoek’s limit and the electromagnetic interference shielding (EMI) efficiency, according to Schelkunoff theory (SE = A + R + B) is the total sum of absorbed, reflected and internal reflected diminution [66]. Figure 7c illustrates the effect of nitrogen gas on PL values for the Ni_72_Fe_18_Ag_10_ composition films deposited at 115 W.

It is clear that with the incorporation of nitrides in thin film samples, the transmission loss is reduced considerably as compared to same composition thin films that are deposited with argon gas alone. The lowest values of 38.74% are observed in sample-8 produced under Ar+N_2_ sputtering gas mixture, with an optimum 20 sccm flow rate of nitrogen. Power absorption of those soft-magnetic thin films is mainly due to material loss and frequency. Besides, the resonance frequency fluctuating from 1.5 to 3 GHz can be regulated by controlling thickness of the film [67]. The maxima of the PL curves in Figure 7c has moved to higher frequency, which is important for their use as EM absorbing materials.

### 3.5. Magnetic Field Coupling Efficiency

**Figure 8:**
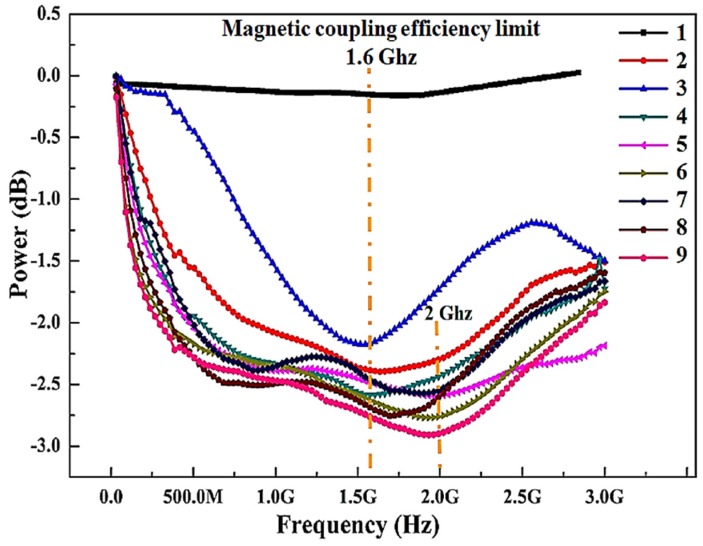
Magnetic coupling efficiency limit dependence on the composition and sputtering parameters of flexible thin film samples.

Keeping in view the application goals, we want to obtain high values of the magnetization along with lower coercivity and power loss in the as deposited thin films, indicating good soft magnetic properties. The results shown in Figure 6 and Figure 7 reveals that selection of proper composition and processing parameters is crucial. The efficiency of magnetic field coupling is shown in Figure 8 by connecting with and without the thin film samples using the transmission coil as a backplane.

By attaching the thin films to the transmission coil as a backplane, a decrease of the inductance is primarily the current and magnetic loss of the sample. As shown in Figure 8, with and without attaching the samples shows the steep change of received power values denoting the magnetic function. Doping of 5% Ag in samples 1–3, shows that the magnetic loss and dielectric loss is significantly higher. Increasing the sputtering power from 100–130 W, the improvement of the received power values proves that increasing thickness of samples 4–6 can reduce the loss of thin films and the magnetic coupling efficiency reaches to a maximum at 1.6 GHz frequency.

Different thickness of thin films leads to the apparent change of received power values, representing the material loss (magnetic loss + dielectric loss) and an enhanced ability to guide the magnetic flux, respectively. With the incorporation of nitrides (samples 7–9), magnetic field coupling efficiency moved towards higher frequencies up to 2 GHz. Sample 8 and 9 attained the lowest values of reducing magnetic loss and dielectric loss. Instead, the better inductance is the ability of attached sample gathering the magnetic field on account of its high M and low Hc, but the geometrical measurements of the coil are directed by the design. Further increase of frequency deteriorates the coupling efficiency, but still remains appreciable. No data has been found in the literature for blue tooth frequency range of 3 GHz. From the above analysis, it is clear that high M and low Hc along with low material loss contributes to the excellent magnetic field coupling efficiency of thin films deposited under Ar+N_2_ gas mixture.

### 3.6. Surface Free Energy (SFE)

**Figure 9:**
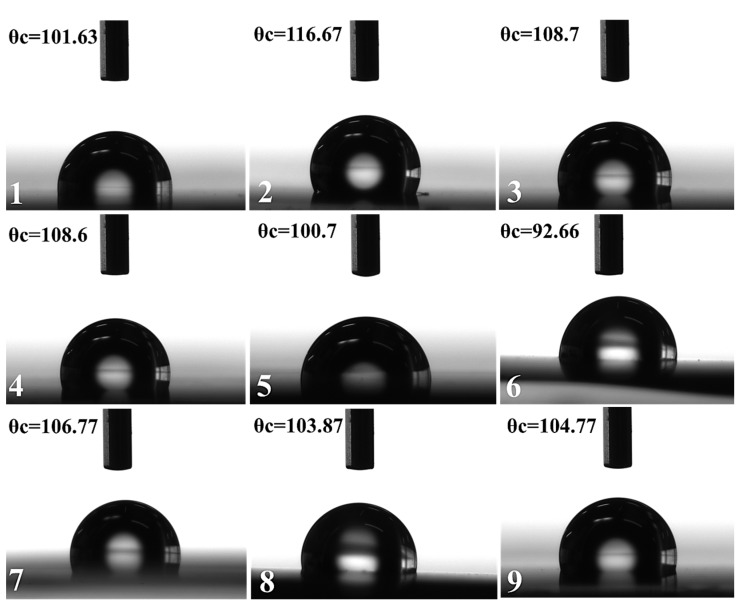
Digital images of contact angle (θc) between thin film samples 1–9 and the polar test liquid water at room temperature.

Figure 9 shows the contact angle (θc) between thin film samples 1–9 and the test liquid water, which is a quantitative measure of wetting characteristic of a thin film surface by any testing liquid [68,69].

Contact angle values for two different testing liquids (water and ethylene glycol) were carefully taken from the digital images of the sessile droplets on the thin films and resultant surface energy of the thin film can be calculated precisely. Acid-Base methods in the equation of states developed by Owens, Wendt, Rabel, and Kaelble were followed to calculate the SFE for silver permalloy thin films [70,71]. We use water as a polar liquid and ethylene glycol as dispersive liquid. The sum of dispersive (γ^d^) and polar (γ^p^) components computes to total surface energy of any thin film, according to the following equation.

(4) $$\gamma_{L}\;\left(1+\cos\theta \right)=2\left\{\sqrt{\gamma_{L\;}^{d}\gamma_{S}^{d}}+\sqrt{\gamma_{L\;}^{p}\gamma_{S}^{p}}\right\}\;\gamma_{L}=\mathrm{Surface}\;\mathrm{free}\;\mathrm{Energy}\;$$

Table 3 shows the calculated surface free energy from measured static contact angles for silver doped permalloy flexible thin films.

According to Table 3, all of the samples have contact angle with water >90°, hence the nature of thin films is hydrophobic. Sample-1 shows the highest surface energy, 80.08 mJ/m^2^, as compared to sample-2 and 3. Due to the increased sputtering power from 100–130 W, the grain size of thin films tend to increase. The mean grain size exceeds within plasma beam is because of thermally driven dynamic grain coarsening. Moreover, as the thickness increases, the grains in thin films become bigger. These results show that the surface energy is inversely proportional to grain size of the samples. In the second set of thin films with higher percentage of silver doping, sample-4 deposited at 100 W shows higher surface energy, 79.97 mJ/m^2^, as compared to sample-5 and 6. Also, the higher kinetic energy will allow for the ad-atoms to possess enhanced transverse mobility, which favors the formation of more crystalline films. Because of the smaller grain size, we achieved lesser film thickness at a lower sputtering power [72]. The decrease of the sputtering power results in the decreased kinetic energy of the sputtered atoms, which makes it difficult for more atoms to reach the substrate. Consequently, a lesser deposition rate and film thickness.

It is interesting to note that nitrogenized Ag-permalloy thin films deposited under Ar+N_2_ gas mixture shows the highest surface energies due to the formation of nitride phase, as depicted in XRD. Sample-7, deposited with 10 sccm of nitrogen flow rate, exhibits surface energy equals to 102.63 mJ/m^2^, and this suggests that even a small amount of nitrogen during sputtering will decrease the grain size. Sample-8 deposited under 15 sccm of nitrogen flow exhibits the maximum SFE, 113.69 mJ/m^2^ amongst all of the samples, indicating that the incorporation of nitrogen in the film helps the growth of smaller crystallites [27]. Nitrogen gas while forming nitride phase also incorporates the interstitial positions in FCC NiFe and restricts the grain growth of the Permalloy. This also causes a slight reduction in the magnetic moment as observed in Figure 6c. Thin films sample-8 will exert a greater self-cleaning effect (stain repellent) than other films, confirming the optimal flow rate of nitrogen at 15 sccm and sputtering power to 115 W. Samples 1, 4, and 8 will have superior adhesion and liquid absorption area. The adhesion is very closely related to SFE. High surface energy corresponds to strong adhesion. Thin films having high surface free energy, shows high liquid-absorption capacity, and the area of the liquid absorption is also higher. There are numerous potential applications for these newly developed thin films. Owing to their effectiveness as a magnetic core material in electrical and electronic equipment, and also in magnetic shielding to block magnetic fields. Nitrogenized Ag-doped permalloy thin films can be wrapped to underwater telecommunication cable to increase the signaling speed, hence the wettability and surface free energy measurements are necessary to assess the in-service compatibility requirements.

### 3.7. (SEM) Structural Morphology

**Figure 10:**
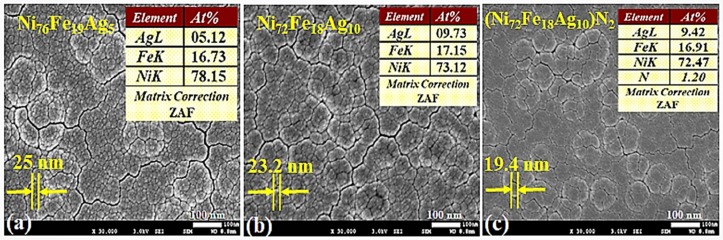
Surface morphology of nanostructured Ag-doped permalloy thin films deposited at 115 W. (**a**) Ni_76_Fe_19_Ag_5_; (**b**) Ni_72_Fe_18_Ag_10_;and (**c**) (Ni_72_Fe_18_Ag_10_)N_2_.

SEM images for the as-deposited thin films on PETE substrate are presented in Figure 10. Samples 2, 5, and 8 are shown in Figure 10a–c, respectively. In order to simplify the understanding, only the samples that are deposited with 115 W sputtering power will be discussed. During the deposition, both temperature and bias parameters were similar for all of the samples.

SEM images in Figure 10 shows the globular microstructure in terms of nano-modules with evident cluster formation at the surface. Within the clusters, nano-sized grains having clear grain boundaries are present homogeneously. The insets shown are averaged EDS analysis, performed at various points of thin films. These results clearly indicate a homogeneous composition across the thin films confirming the XPS analysis.

Thin film images in Figure 10a,b showing an average grain size of 25.0 nm and 23.2 nm respectively, deposited under Ar gas only. Surface of sample-b, looks smoother owing to the change in chemical composition and this is generally anticipated for the films produced by magnetron sputtering [73,74]. With nano-grain features, films exhibit uniform surface topographies that validate their nanoscale character. Film deposition conditions, such as gas mixture, flow rate, and sputtering powers become key factors that govern the film growth. Figure 10c shows the finer average grain size of 19.4 nm, owing to the effect of optimal nitrogen flow rate used in the Ar+N_2_ gas mixture at 115 W during deposition. Geometric shadowing during film deposition favors the “gain”-like morphology, where some regions attracts more atoms and hinder the deposition flux to lower regions [75]. During the deposition, both temperature and bias parameters were similar for all of the samples. Further work is in progress to perform AFM for surface roughness, EIS for corrosion response, and mechanical testing to check the flexibility limits of thin films.

## 4. Conclusions

Silver-permalloy thin films possessing tunable functional properties were successfully deposited on flexible substrate by magnetron sputtering. Average film deposition rate was confirmed at around 2.05 nm/min for argon atmosphere and reduced to 1.8 nm/min in reactive nitrogen atmosphere. The grain size for the film sputtered with Ar only was 23–25 nm, which decreases after sputtering with nitrogen. Nitrogenized Ag-Permalloy thin film reveals that the incorporation of nitrogen favors the growth of smaller crystallites size of ±19 nm, and no loss features were observed in XPS spectra showing good stability of thin films. Stoichiometry was confirmed to be intact in XPS and EDS analysis, indicating that the flexible thin film samples remains as silver-doped Permalloy. As the sputtering power is increased from 100–130 W for thin films grown in Ar atmosphere, the broadening of reflection and shift toward higher angle is observed. Moreover, the occurrence of two peaks of paramagnetic (Fe_3_Ni) N, as determined by XRD, leads to the increase of coercivity. It is also observed that the reduction of the average crystallite size, as well as the presence of small amount of paramagnetic phase, causes an important change in the magnetization reversal mechanism. As the nitrogen partial pressure is increased, broadening of the X-ray diffraction (210) peak is a clear indication of the nanocrystalline nature of the films because incorporation of nitrogen favors the growth of smaller crystallites. The higher intensity of an Ag phase peak is due to the (111) preferred orientation and the non-fluorescent nature of FeNi and (Fe_3_Ni) N phases. All of the samples show a globular microstructure in terms of nano-modules with evident cluster formation at the surface. Within the clusters, nano-sized grains having clear grain boundaries are present homogeneously. Best soft magnetic properties (M = 74.59 emu/g, Hc = 6.81 Oe) were exhibited by thin film deposited under argon gas alone at 130 W. While thin film deposited under reactive nitrogen atmosphere at 115 W shows comparable magnetic performance, lowest magnetic loss, and highest surface free energy, confirming that 15 sccm flow rate is optimal for producing Ag-doped permalloy flexible thin films having excellent magnetic field coupling efficiency.

## Figures and Tables

**Table 1:** Thin film deposition processing parameters for two different target compositions.

Sample #	Target Composition	Gas Flow Rate (SCCM)	Sputtering Power (W)	Film Thickness (nm)	Deposition Rate (nm/min)
1	**Ni_76_Fe_19_Ag_5_**	Ar = 60	100	180	1.8
2			115	192	1.92
3		″	130	236	2.36
4	**Ni_72_Fe_18_Ag_10_**	″	100	185	1.85
5		″	115	196	1.96
6		″	130	242	2.42
7		N_2_ = **10 +** Ar = 60	115	198	1.8
8		N_2_ = **15 +** Ar = 60	115	205	1.86
9		N_2_ = **20 +** Ar = 60	115	208	1.89

**Table 2:** Binding energies, separation values, atomic sensitivity factor (ASF), oxidation state, and chemical composition during XPS.

Element	Binding Energy/s (B.E) eV	Separation (Δ) eV	Atomic Sensitivity Factor (ASF [56])	Oxidation State	Composition (at %) Samples		
					1–3	4–6	7–9
Ni	2p_3/2_ = 873.7 2p_1/2_ = 857.3	16.4	3.7	Ni^2+^	77.9	73.2	72.5
Fe	2p_3/2_ = 711.7 2p_1/2_ = 723.8	12	2.7	Fe^2+^ Fe^3^^+^	16.9	17.1	17
Ag	3d_5/2_ = 367.5~368.3 3d_3/2_ = 373~374	5.5~5.6	5.2	Ag^3d^	5	9.7	9.3
N	N1s = 400.9	-	0.48	-	-	-	1.2

**Table 3:** Surface free energy calculated from data of measured contact angle of silver-doped permalloy flexible thin films.

Target Composition	Sample	Contact Angle (θc)		Polar Component (γ_s_^p^)	Dispersive Component (γ_s_^d^)	Surface Free Energy (mJ/m^2^)
		Water	Ethylene Glycol			
Ni_76_Fe_19_Ag_5_	**1**	**101.63**	**48.13**	**77.27**	**2.815**	**80.08**
	2	116.67	73	54.732	4.106	58.84
	3	108.7	71.74	42.05	3.60	45.65
Ni_72_Fe_18_Ag_10_	**4**	**108.6**	**56.84**	**75.16**	**4.816**	**79.97**
	5	100.7	49.43	71.98	1.942	73.93
	6	92.66	50.3	52.09	0.020	52.11
(Ni_72_Fe_18_Ag_10_)N_2_	7	106.77	51.09	95.08	7.553	102.63
	**8**	**103.87**	**37.56**	**105.60**	**8.089**	**113.69**
	9	104.77	47.06	88.04	5.462	93.50
